# Proposal of a new nomenclature for introns in protein-coding genes in fungal mitogenomes

**DOI:** 10.1186/s43008-019-0015-5

**Published:** 2019-10-10

**Authors:** Shu Zhang, Yong-Jie Zhang

**Affiliations:** 1 Institute of Applied Chemistry, Shanxi University, Taiyuan, 030006 China; 2 School of Life Science, Shanxi University, Taiyuan, 030006 China

**Keywords:** Nomenclature, *Fungi*, Mitogenome, Intron, Protein-coding gene

## Abstract

Fungal mitochondrial genes are often invaded by group I or II introns, which represent an ideal marker for understanding fungal evolution. A standard nomenclature of mitochondrial introns is needed to avoid confusion when comparing different fungal mitogenomes. Currently, there has been a standard nomenclature for introns present in rRNA genes, but there is a lack of a standard nomenclature for introns present in protein-coding genes. In this study, we propose a new nomenclature system for introns in fungal mitochondrial protein-coding genes based on (1) three-letter abbreviation of host scientific name, (2) host gene name, (3), one capital letter P (for group I introns), S (for group II introns), or U (for introns with unknown types), and (4) intron insertion site in the host gene according to the cyclosporin-producing fungus *Tolypocladium inflatum*. The suggested nomenclature was proved feasible by naming introns present in mitogenomes of 16 *fungi* of different *phyla*, including both basal and higher fungal lineages although minor adjustment of the nomenclature is needed to fit certain special conditions. The nomenclature also had the potential to name plant/protist/animal mitochondrial introns. We hope future studies follow the proposed nomenclature to ensure direct comparison across different studies.

**Electronic supplementary material:**

The online version of this article (10.1186/s43008-019-0015-5) contains supplementary material, which is available to authorized users.

## INTRODUCTION

*Fungi* constitute a huge group of highly diverse organisms, with 2.2–3.8 million estimated species and 144,000 currently known species on Earth (Hawksworth and Lücking 2017; Cannon et al. 2018). They were traditionally divided into four groups: chytridiomycetes, zygomycetes, ascomycetes, and basidiomycetes according to morphological traits associated with reproduction. Molecular phylogenetics and more recently phylogenomics recognized eight *phyla* in *Fungi*, namely *Microsporidia*, *Cryptomycota*, *Blastocladiomycota*, *Chytridiomycota*, *Zoopagomycota*, *Mucoromycota*, *Ascomycota*, and *Basidiomycota* (Spatafora et al. 2017). Aside from a few early divergent lineages and anaerobic organisms, almost all *fungi* contain mitochondria and mitogenomes in their cells (Bullerwell and Lang 2005; van der Giezen et al. 2005). Over recent years, mitogenomes of an increasing number of fungal species are sequenced. As of July 2019, mitogenomes from at least 300 fungal species are available with representatives from all major fungal groups. Fungal mitogenomes typically contain 15 standard protein-coding genes, two rRNA genes and a variable number of tRNA genes. These protein-coding genes are *atp6*, *atp8*, *atp9*, *cob*, *cox1*, *cox2*, *cox3*, *nad1*, *nad2*, *nad3*, *nad4*, *nad4L*, *nad5*, *nad6*, and *rps3* (Lang 2018), and some of them may be absent from certain fungal mitogenomes (Koszul et al. 2003).

Introns as mobile elements are frequently observed in mitochondrial protein-coding and/or rRNA genes of *fungi*. One gene may also be simultaneously invaded by multiple introns (e.g., four introns in *cob* and seven introns in *cox1* in *Isaria cicadae*) (Fan et al. 2019). Mitochondrial introns are divided into two groups (I and II) based on their secondary structure and splicing mechanism (Saldanha et al. 1993), with group I introns being abundant in fungal mitogenomes. Different fungal species or even different individuals of a particular fungus may show diversity in number and insertion position of mitochondrial introns (Kosa et al. 2006; Zhang et al. 2015; Zhang et al. 2017a; Wang et al. 2018; Fan et al. 2019; Nie et al. 2019). Introns contribute to fungal mitogenome expansion/variability and represent an ideal marker for understanding fungal evolution (Zhang et al. 2015).

Currently, there has been a nomenclature for introns present in rRNA genes (Johansen and Haugen 2001). According to the nomenclature, introns are often found at a limited number of insertion sites in highly conserved regions of rRNA genes from nuclei, mitochondria, and chloroplasts, and therefore, a given rRNA sequence can be aligned with the chosen standard rRNA sequences of *Escherichia coli* to locate and name potential introns. For mitochondrial protein-coding genes, however, it is difficult to align their sequences with corresponding *E. coli* sequences due to high sequence divergence. In most literatures, introns in protein-coding genes are generally named serially according to their appearance in a particular host gene (e.g., *cox1*-i1, *cox1*-i2, and *cox1*-i3) (Deng et al. 2016; Zhang et al. 2017b; Zhang et al. 2017c). This naming strategy is not convenient for scientific communication and comparison of introns across different mitogenomes. A standard nomenclature of mitochondrial introns is needed to avoid confusion when comparing different fungal mitogenomes.

In our previous studies, we have tried to designate introns based on their insertion positions, but a mitogenome is arbitrarily selected from species under investigation (Fan et al. 2019; Zhang et al. 2019). In this study, we aim to propose a standard nomenclature for introns in protein-coding genes in fungal mitogenomes and test its applicability using fungal species from a broad range of taxonomic classification. To know if the suggested nomenclature can apply to “cross-*kingdom*” mitochondrial introns, some plant/protist/animal introns are also examined.

## METHODS

In order to establish a standard nomenclature for introns in protein-coding genes across the *kingdom* *Fungi*, it is necessary to find an appropriate reference mitogenome. By looking at fungal species with available mitogenomes, we choose the mitogenome of the cyclosporin-producing fungus *Tolypocladium inflatum* ARSEF 3280 (accession number NC_036382) as the reference mitogenome. The 25,328-bp mitogenome of *T. inflatum* contains all the 15 protein-coding genes typically found in fungal mitogenomes, and there is no intron in any of these protein-coding genes (Zhang et al. 2017d). We did not choose the best-understood model *fungi*: ‘baker’s yeast’ *Saccharomyces cerevisiae*, the fission yeast *Schizosaccharomyces pombe*, the opportunistic fungal pathogen *Candida albicans*, the filamentous euascomycete *Neurospora crassa*, etc. This is because the yeasts *Sa. cerevisiae* and *Sc. pombe* both lack genes coding for NADH dehydrogenases in their mitogenomes (Foury et al. 1998), and *C. albicans* and *N. crassa* contain introns in many different protein-coding genes (Borkovich et al. 2004; Bartelli et al. 2013). We also did not choose the human mitochondrial genome, which was selected as the reference to name introns found in *nad5* and *cox1* in certain metazoans (Emblem et al. 2011). This is because the human mitogenome contains only 13 standard protein-coding genes without *atp9* and *rps3*. The latter two genes are known to harbor introns in fungal mitogenomes.

Both basal and higher *fungi* may contain introns in their mitogenomes. We randomly selected representative species in each fungal phylum to locate and name possible introns (Table 1). Determination of the insertion position of an intron relies on alignment between sequences of its host gene and corresponding gene sequences of *T. inflatum* (Additional file 1). Although there are many sequence alignment programs available, we recommend using MAFFT (https://mafft.cbrc.jp/alignment/software/), which is fast when aligning long sequences containing many introns and can always generate satisfactory alignment according to our experience. The default setting of MAFFT works well in most cases. If exon-intron boundaries are not correctly identified (probably due to the interference of intron sequences or presence of short exons) under the default settings, one may consider adjusting the alignment parameters (e.g., try ‘Unalignlevel > 0’ and possibly ‘Leave gappy regions’ by selecting the G-INS-1 or G-INS-i alignment strategy) and/or importing additional sequences to align from a species closely related the test species. In addition, it is always advisable to refer to known annotation results and/or characteristic nucleotides at splice sites of group I/II introns (Cech 1988) to ensure correct alignment and identification of exon-intron boundaries.

**Table 1:** Selected fungal species and their mitogenome information

Fungal taxa	Accession	Length (nt)	Phylum	Class	Order	Family	Code
Basal *fungi*							
*Rozella allomycis*	NC_021611	12,055	*Cryptomycota*				4
*Rhizopus oryzae*	NC_006836	54,178	*Mucoromycota*		*Mucorales*	*Rhizopodaceae*	1
*Conidiobolus heterosporus*	MK049352	53,364	*Zoopagomycota*	*Entomophthoromycetes*	*Entomophthorales*	*Ancylistaceae*	4
*Allomyces macrogynus*	NC_001715	57,473	*Blastocladiomycota*	*Blastocladiomycetes*	*Blastocladiales*	*Blastocladiaceae*	4
*Hyaloraphidium curvatum*	NC_003048	29,593	*Chytridiomycota*	*Monoblepharidomycetes*	*Monoblepharidales*		4
Higher *fungi*							
*Candida albicans*	NC_002653	40,420	*Ascomycota*	*Saccharomycetes*	*Saccharomycetales*	*Debaryomycetaceae*	4
*Grosmannia piceiperda* ^a^	FJ717837	2928	*Ascomycota*	*Sordariomycetes*	*Ophiostomatales*	*Ophiostomataceae*	
*Isaria cicadae*	MH922223	56,581	*Ascomycota*	*Sordariomycetes*	*Hypocreales*	*Cordycipitaceae*	4
*Neurospora crassa*	NC_026614	64,840	*Ascomycota*	*Sordariomycetes*	*Sordariales*	*Sordariaceae*	4
*Saccharomyces cerevisiae*	NC_001224	85,779	*Ascomycota*	*Saccharomycetes*	*Saccharomycetales*	*Saccharomycetaceae*	3
*Schizosaccharomyces pombe*	NC_001326	19,431	*Ascomycota*	*Schizosaccharomycetes*	*Schizosaccharomycetales*	*Schizosaccharomycetaceae*	4
*Tolypocladium inflatum*	NC_036382	25,328	*Ascomycota*	*Sordariomycetes*	*Hypocreales*	*Ophiocordycipitaceae*	4
*Cryptococcus neoformans*	NC_004336	24,874	*Basidiomycota*	*Tremellomycetes*	*Tremellales*	*Tremellaceae*	4
*Puccinia striiformis*	NC_039655	101,521	*Basidiomycota*	*Pucciniomycetes*	*Pucciniales*	*Pucciniaceae*	4
*Tilletia indica*	NC_009880	65,147	*Basidiomycota*	*Exobasidiomycetes*	*Tilletiales*	*Tilletiaceae*	4
*Tricholoma matsutake*	NC_028135	76,037	*Basidiomycota*	*Agaricomycetes*	*Agaricales*	*Tricholomataceae*	4

^a^ For *Grosmannia piceiperda*, only sequences of the *rnl* gene are known. In this species, an *rnl* group IA intron (mL2449) encodes an *rps3* gene which is further fragmented by the insertion of a group IC2 intron (Rudski and Hausner 2012)

## RESULTS AND DISCUSSION

We propose a new nomenclature system for introns in fungal mitochondrial protein-coding genes based on (1) three-letter abbreviation of host scientific name, (2) host gene name, (3) one capital letter P (for group I introns, meaning position or primary for easy memorization), S (for group II introns, meaning site or secondary), or U (for introns with unknown types), and (4) intron insertion site in the host gene according to *T. inflatum* (Additional file 1). When there is no ambiguity (e.g., when just talking about introns in a particular species or in a particular host gene of a species), host scientific name and/or host gene name may be omitted. In any case, however, the letter P/S/U and insertion site of an intron should never be omitted. Using the nomenclature, previously reported introns could be renamed. Examples of renaming are the group II intron Sce.cox1S169 (former aI1) from *Saccharomyces cerevisiae cox1* at site 169, and the group I intron Cgl.cox1P240 (former CgCox1.1) from *Candida glabrata cox1* at position 240. Other examples are included in Table 2 (lines 1–10). We hope future studies follow this proposed nomenclature to ensure direct comparison across different studies.

**Table 2:** Representative examples of the new nomenclature of introns in protein-coding genes ^a^

Line	New name	Old name	Fungal taxa	Host gene	Accession	Note	Reference
1	Sce.cox1S169	aI1	*Saccharomyces cerevisiae*	*cox1*	NC_001224	Group II intron	Foury et al. 1998
2	Sce.cox1P971	aI5α	*Saccharomyces cerevisiae*	*cox1*	NC_001224	Group I intron	Foury et al. 1998
3	Sce.cox1P1107	aI5β	*Saccharomyces cerevisiae*	*cox1*	NC_001224	Group I intron	Foury et al. 1998
4	Sce.cox1S1132	aI5ɣ	*Saccharomyces cerevisiae*	*cox1*	NC_001224	Group II intron	Foury et al. 1998
5	Cgl.cox1P240	CgCox1.1	*Candida glabrata*	*cox1*	NC_004691	Group I intron	Koszul et al. 2003
6	Cgl.cox1P386	CgCox1.2	*Candida glabrata*	*cox1*	NC_004691	Group I intron	Koszul et al. 2003
7	Cgl.cox1P971	CgCox1.3	*Candida glabrata*	*cox1*	NC_004691	Group I intron	Koszul et al. 2003
8	Cme.cobP393	bI1	*Candida metapsilosis*	*cob*	NC_006971	Group I intron	Kosa et al. 2006
9	Hth.nad1P636	nad1-i1	*Hirsutella thompsonii*	*nad1*	NC_040165	Group I intron	Wang et al. 2018
10	Ici.atp9P181	atp9-i1	*Isaria cicadae*	*atp9*	MH922223	Group I intron	Fan et al. 2019
11	Cpse.nad5U717		*Candida pseudojiufengensis*	*nad5*	NC_022156	Unknown intron type	Unpublished
12	Cpsy.nad5P717		*Candida psychrophila*	*nad5*	NC_036103	Group I intron	Unpublished
13	Hau.cox3P640a	cox3-i2	*Hypomyces aurantius*	*cox3*	NC_030206	1st one in twintron	Deng et al. 2016
14	Hau.cox3P640b	cox3-i2	*Hypomyces aurantius*	*cox3*	NC_030206	2nd one in twintron	Deng et al. 2016
15	Hth.cobP429–1	cob-i2	*Hirsutella thompsonii*	*cob*	NC_040165	Strain: ARSEF 9457	Wang et al. 2018
16	Hth.cobP429–2	cob-i2	*Hirsutella thompsonii*	*cob*	MH367296	Strain: ARSEF 1947	Wang et al. 2018
17	Zsa.nad5P717	ND5–717	*Zoanthus sansibaricus*	*nad5*	KY888672	Coral: Group I intron	Chi and Johansen 2017
18	Zsa.cox1P867	COI-867	*Zoanthus sansibaricus*	*cox1*	KY888672	Coral: Group I intron	Chi and Johansen 2017
19	Mbr.nad5P717		*Monosiga brevicollis*	*nad5*	AF538053	Protist: Group I intron	Burger et al. 2003
20	Ddi.cox2P357		*Dictyostelium discoideum*	*cox1/2*	NC_000895	Protist: Group I intron	Ogawa et al. 2000
21	Mpo.nad5P717		*Marchantia polymorpha*	*nad5*	M68929	Plant: Group I intron	Oda et al. 1992
22	Ath.cox2S691		*Arabidopsis thaliana*	*cox2*	NC_037304	Plant: Group II intron	Sloan et al. 2018

^a^ Examples from lines 1 to 16 are fungal species, and those from lines 17 to 22 are plant/protist/animal species as indicated in the column “Note”

The suggested nomenclature is flexible to fit some special conditions. Firstly, although we suggest three-letter abbreviation of host scientific name, four-or-more-letter abbreviation may be used in cases where the three-letter abbreviation cannot discriminate among all species under investigation. An example is introns at position 717 in *nad5* in *Candida pseudojiufengensis* (Cpse.nad5U717) and *Candida psychrophila* (Cpsy.nad5P717) (Table 2, lines 11–12). Secondly, twintrons (twin introns) have been described from some fungal mitogenomes with various combinations of group I or II introns nested inside each other or situated next to each other (Hafez and Hausner 2015; Deng et al. 2016). The internal/external or upstream/downstream members of a twintron could be named alphabetically. An example is the side-by-side twintron in *cox3* in *Hypomyces aurantius*, where two group IA introns are arranged in tandem (Deng et al. 2016). The upstream intron of the twintron can be named as Hau.cox3P640a and the downstream one as Hau.cox3P640b (Table 2, lines 13–14). Finally, although introns present at an identical insertion site among different strains of a particular species are generally conserved, distantly related introns are sometimes detected among different strains. Introns of this kind can be named numerically. For example, Hth.cobP429 in different strains of *Hirsutella thompsonii* showed length variations (e.g., 2.7 kb in ARSEF 9457 and 4.8 kb in ARSEF 1947) (Wang et al. 2018), and the two variants may be named as Hth.cobP429–1 in ARSEF 9457 and Hth.cobP429–2 in ARSEF 1947 (Table 2, lines 15–16).

The suggested nomenclature has been successfully applied to name introns in 16 *fungi* from different *phyla*, including both basal and higher fungal lineages (Table 3). These *fungi* contain introns in all protein-coding genes except *atp8*, *nad2*, and *nad6*, and *cob* and *cox1* are most frequently invaded by introns. These introns are mostly group I introns, but we also find few group II introns as well as few introns with undetermined types. There are a total of 149 introns at 74 insertion sites in these *fungi*. Using the suggested nomenclature, intron positions in a particular gene can be directly observed and compared across different species. We find some points frequently inserted by introns in different species (e.g., cobP490, cox1P386, cox1P720, cox1P1107). From the intron insertion site numbers, one can also easily understand the phase of an intron, which is phase 0 when an intron inserts between two codons (e.g., cobP393), and phase 1 or 2 when an intron inserts within a codon (e.g., cox1S205, cox1P386). These introns are often found at highly conserved regions (Additional file 2).

**Table 3:** Intron positions in mitochondrial protein-coding genes of selected fungal species ^a^

Fungal taxa	*atp6*	*atp8*	*atp9*	*cob*	*cox1*	*cox2*	*cox3*	*nad1*	*nad2*	*nad3*	*nad4*	*nad4L*	*nad5*	*nad6*	*rps3*	No. introns	No. genes with introns
*Cryptomycota*																	
*Rozella allomycis*	0	-- ^b^	0	0	P731	0	0	–	–	–	–	–	–	–	–	1	1
*Mucoromycota*																	
*Rhizopus oryzae*	0	0	P157	P393, P490	P386, P615, P720	P685	P219	0	0	P124	0	0	0	0	–	9	6
*Zoopagomycota*																	
*Conidiobolus heterosporus*	U374	0	P69	P201, P393, P429, P506, P600, P759, P823	P212, P281, P386, P461, P550, P615, P720, P731, P807, P867, P1107, P1125, P1305	P228, P328	P447	U166, P636	0	0	P915	0	P426, U1059	0	0	30	9
*Blastocladiomycota*																	
*Allomyces macrogynus*	0	0	0	P201, P417, P429, P490, P600, P759	P221, P281, S313, P372, P386, P615, P678, P867, P1030, P1107, P1230, P1296	P685	0	P166, P636	0	0	0	0	P426, P717, P934	0	0	24	5
*Chytridiomycota*																	
*Hyaloraphidium curvatum*	0	0	0	P411	0	0	0	0	0	0	0	0	0	0	–	1	1
*Ascomycota*																	
*Candida albicans*	0	0	0	U393, U429	P386, P709, P720, P1107	0	0	0	0	0	0	0	0	0	–	6	2
*Grosmannia piceiperda*	NA ^c^	NA	NA	NA	NA	NA	NA	NA	NA	NA	NA	NA	NA	NA	P159	1	1
*Isaria cicadae*	U572	0	P181	P393, P490, P506, P823	P212, P281, P709, P720, P731, P1057, P1281	P228, P357, P651	P219, P631	P636	0	0	0	0	P417, P570	0	0	21	8
*Neurospora crassa*	P344	0	0	P393, P490	0	0	0	P636	0	P90	P505	P263	P324, P717	0	0	9	7
*Saccharomyces cerevisiae*	0	0	0	S415, P429, P506, P759, P809	S169, S205, P240, P720, P971, P1107, S1132	0	0	–	–	–	–	–	–	–	0	12	2
*Schizosaccharomyces pombe*	0	0	0	S687	P386, P731	0	0	–	–	–	–	–	–	–	–	3	2
*Tolypocladium inflatum*	0	0	0	0	0	0	0	0	0	0	0	0	0	0	0	0	0
*Basidiomycota*																	
*Cryptococcus neoformans*	0	0	0	P490	0	0	0	P166	0	0	0	0	0	0	0	2	2
*Puccinia striiformis*	0	0	0	U429, P809	P386, U615, P709, P720, P807, U1107	P314	0	0	0	0	S495	0	P417, P717	0	0	12	5
*Tilletia indica*	0	0	0	P490	P212, P386, P709, P1107, P1305	0	0	0	0	0	0	0	P417, P717	0	–	8	3
*Tricholoma matsutake*	0	0	0	P823	U281, P372, P386, P720, P900	P318, P357	0	P276	0	0	0	0	P417	0	0	10	5
No. introns	3	0	3	35	65	10	4	8	0	2	3	1	14	0	1	149	
No. intron-containing species	3	0	3	13	11	6	3	6	0	2	3	1	7	0	1		
No. intron insertion points	3	0	3	13	29	7	3	3	0	2	3	0	7	0	1	74	

^a^ Re-annotation was performed if online or published annotations failed to correctly identify introns. Intron types were determined by RNAweasel (http://megasun.bch.umontreal.ca/RNAweasel)

^b^ “--” indicates the absence of corresponding genes in a particular mitogenome

^c^ NA, not available

In addition to *fungi*, plants and protists (but rarely in animals) also contain group I or II introns in their mitochondrial genes (Oda et al. 1992; Ogawa et al. 2000; Burger et al. 2003; Chi and Johansen 2017). The nomenclature suggested in this study could potentially apply to plant/protist/animal mitochondrial introns (Table 2, lines 17–22; Additional file 2). Plant mitogenomes, however, are also known to encode several intron-containing protein genes (e.g., *nad7*, *ccmC*, *rps10*, *rpl2*) that are absent in fungal mitogenomes (Zhang et al. 2011; Sloan et al. 2018). Introns are even found in tRNA-coding genes in plant mitogenomes (Smith et al. 2011). An additional plant reference is necessary to name introns unique to plant mitogenomes.

## CONCLUSIONS

A standard nomenclature was suggested for introns in protein-coding genes in fungal mitogenomes. It was proved feasible by naming introns present in mitogenomes of 16 *fungi* from a broad range of taxonomic classification, and it also had the potential to name introns in plant/protist/animal mitogenomes. Future studies should follow the proposed nomenclature to ensure direct comparison across different studies.

## Additional files

Additional file 1: Sequences of protein-coding genes of *Tolypocladium inflatum* ARSEF 3280 (accession number NC_036382). Insertion site of group I introns are shown in red, group II introns in green, and introns with undetermined intron types in shade. (DOCX 21 kb)

Additional file 2: Intron insertion sites for 22 common introns. Exon sequences of *cob*, *cox1*, *cox2*, *nad1*, and *nad5* of different fungal taxa plus few non-fungal taxa were aligned by MAFFT, and visualization of the aligned sequences was performed using ESPript 3.0 (Robert and Gouet 2014) under default settings. Refer to Tables 1 and 2 for organisms represented by accession numbers, and the accession numbers of non-fungal taxa are marked in red boxes. Insertion sites of introns are shown using upward arrows. For phase 0 introns, conserved amino acids before and after insertion sites are listed. The amino acid glycine (G) is frequently seen before insertion sites of phase 0 introns. For phase 1 or 2 introns, conserved amino acids at insertion sites are given, and corresponding triplet codons are marked by a horizontal line. (PPTX 2235 kb)

## Data Availability

All data used in this study are publicly available.
