# Correction to: Proposal of a new nomenclature for introns in protein-coding genes in fungal mitogenomes

**DOI:** 10.1186/s43008-020-00030-2

**Published:** 2020-03-23

**Authors:** Shu Zhang, Yong-Jie Zhang

**Affiliations:** 1 Institute of Applied Chemistry, Shanxi University, Taiyuan, 030006 China; 2 School of Life Science, Shanxi University, Taiyuan, 030006 China

**Correction to: IMA Fungus (2019) 10:15**

**https://doi.org/10.1186/s43008-019-0015-5**

The published article (Zhang and Zhang 2019) includes an incorrect sequence. The erroneously indicated nad4L sequence NC_001715 (GenBank accession number) should be replaced by NC_036382 (GenBank accession number) in additional file 1.

- Incorrect sequence:

">NAD4L_NC_001715

ATGCTTTTAGAAATAATAACAGCTTATAAAATAGGAACAATCTTATTTTTAATTGGAATTTTAGGTTTCATTATCAATAGACAAAATATTCTTTTACTTATTATCTCTATTGAAATGACTTTATTAGCTATTAGTTTTATTATTATTTGTTCTGCTCTTTTCCTTGATGATTCTGCAGCAGCTTGTTTTTCACTTTATATTTTAGCTCTTGCTGGTTCAGAAGCTGCAATTGGTCTTTCACTTTTAGTTTTATTCCATAGATTTAGAGGATCAGTATTAATTTCAGCTTCTCGACAATAG"

- Correct sequence:

">nad4L_NC_036382

ATGAGTTTAACTTTAGTACTTTTTTTAATAGGAATCTTAGGATTCGTATTTAATAGAAAAAATATAATATTAATGCTTATTTCTATAGAAATAATGCTATTATCTATAACATTTTTAATATTGGTAAGTTCTATTAATCTTGACGATATAATAGGACAAACATATGCTATATACATTATAGTAGTTGCTGGTGCAGAATCTGCTATCGGTTTAGCTATTTTAGTAGCTTTTTATAGACTAAGAGGAAGTATCGCAATAGAATATAAATAA"

To be in concordance with this change, “P263” in the nad4L column should be changed to “P239” in Table 3.

The authors would like to apologise for any inconvenience caused.
