# Supplementary material for: Proposal of a new nomenclature for introns in protein-coding genes in fungal mitogenomes
Source: IMA Fungus. 2019 Oct 10;10:15. doi: 10.1186/s43008-019-0015-5 (PMC7325650; doi:10.1186/s43008-019-0015-5)
Supplement: Supplementary file 1 — Sequences of protein-coding genes of Tolypocladium inflatum ARSEF 3280 (accession number NC_036382). Insertion site of group I introns are shown in red, group II introns in green, and introns with undetermined intron types in shade. (DOCX 21 kb) [file 43008_2019_15_MOESM1_ESM.docx]

>ATP6_NC_036382

ATGTTTACACAAAATTTATTTACAGAAATAAGAAGTCCCTTAGATCAATTTGAAATAAGAGACATATTAAATTTGGAAGTTTTAGGTGGTAACTTACATCTATCTCTAACAAATATAGGATTCTACTTAACATTAGGTTTCTTAATAACATTAATATTAAGTTTAGTTGCAACTAATTATAACAAATTAGTAAGTAACAACTGATCTATAGCTCAAGAATCTTTATACGTAACAATACATAACATAGTTACAAACCAAATAAATGCTAGAAATGGACAAGTTTATTTCCCATTTATATATACATTATTTGTATTTATATTAATTAATAATTTAATCGGTATGATACCTTATAGCTTTGCTTCAACAGGTCATT**T**TGCTTTAACATTTGCTCTTAGTTTCACAATAGTATTAGGAGCAACAATCTTAGGATTCCAAAAACATGGTTTAAAATTCTTTTCATTATTAGTACCAGCTGGTTGTCCTTTAGGTCTTTTACCATTATTAATAACTATAGAATTTATTTCATATTTAGCAAGAAATGTTTCATTAGGACTTAGATTAGCTGCTAATA**T**AACAGCGGGTCACATGTTGTTAAGTATCTTAAGTGGGTTTGTTTATAACATAATGAATTCAGGTATAATATTCTTCGTATTAGGATTAATACCTTTATTATTTATTGTAGCATTCTCAGGATTAGAATTTGCTATAGCATTTATACAAGCACAAGTATTTGTAGTATTATCATCTTCATATATAAAAGATGGATTAGATTTACATTAA

>ATP8_NC_036382

ATGCCACAATTAACACCTTTTTATTATATGAATGAAATAGTATTTGCTTTTGCTGTAATAGTATTAGTATTATACATATTATCTAAATACATATTACCAAGAATAGTTCGTTTATTCTTATCACGTATGTTTATTAATAAAATATAA

>ATP9_NC_036382

ATGTTACAATCATCAAAAATAATAGGAGCAGGATTAGCTACAGTTGGAGTTTTAGGAGCTGGAGTAGGAATAGGAGTAGTATTCGGAGGTTTAATCTTAGGTGTTGCTAGAAACCCTTCATTAAAAAACCAATTATTCTCTTATGCTATATTAGGGTTTGCTTTCTCAGAAGCTACAGCTTTATTCGCTTTAATGATGTCTTTACTTTTATTATACGTTGCTTAG

>COB_NC_036382

ATGAGATTATTAAAAAGTCATCCTTTCTTAAAATTAGTTAATGCTTACGTAATAGATCATTCACAACCAAGTAATATAAATTACTTATGAAATTTCGGTTCTTTATTAGGACTTTGTTTAGGTATACAAATAGTTACAGGTGTAACTTTAGCTATGCACTATAACCCTAGTATAGCCGAAGCATTCAATTCTGTTGAACACATAATGCGTGATGTAAATAACGGATGATTAGTTCGTTACTTACACAGTAATACAGCCTCAGCTTTCTTCTTCTTAGTGTACTTACACATAGGTAGAGGTTTCTACTACGCATCTTACAGAGCTCCTAGAACCTTAGCCTGAACTATAGGTGTGGTAATTCTTATTCTTATGATAGGAACAGCTTTCTTAGGATACGTACTTCCATATGGGCAAATGTCATTATGAGGTGCTACAGTTATTACTAATTTAATTAGTGCTATACCATGAATAGGACAAGATATAGTTGAATTCATCTGAGGTGGATTCTCTGTTAATAACGCTACTTTAAACAGATTCTTTGCTTTACACTTCGTGTTACCTTTTGTATTAGCTGCATTAGTATTAATGCACTTAATAGCAGTACATGATACAGCTGGAGCAAGTAATCCTTTAGGAGTACCAGGATATTATGATAGAATACCTTTCGCACCTTACTACTTATTTAAAGATTTAATTACTATATTTATCTTCTTCTTTGTTTTAAGTATGTTCGTATTCTTCATGCCTAATGTATTAGGTGATAGTGATAATTATATAATGGCTAATCCTATGCAAACACCTGCTGCTATAGTACCAGAATGATACTTATTACCATTCTATGCTATACTGAGATCTATACCTAATAAATTATTAGGGGTTATAGCTATGTTTAGTGCTTTAGTTATAGTATTATTATTACCTAAAGTAGATTTAGGTTTAACTAAAGGTTTACAATTCAGACCTTTAAGTAAAATAATGTTTTATATATTTGTTATAAATTTCTTAATATTAATGCAATTAGGAGCTAAACACGTTGAAAGTCCATTTATAGAATTTGGACAAATCAGTACAGTTCTATATTTCTCTTACTTCTTAGTTATAATGCCTGTTGTAAGTATAATAGAAAATACTTTAATAGATTTATATCAAGTTAATAATAAAGCAAAATAA

>COX1_NC_036382

ATGGGTATGGAAAGATGATTTAATTCAACTAACGCTAAAGATATAGGAACTTTATATTTAATATTTGCTCTATTTTCAGGGTTATTAGGAACAGCAATGTCAGTACTAATAAGACTAGAACTTAGTGGACCAGGAGTTCAATTTATATCAAATAACCAATTATACAACAGTATCGTTACAGCTCACGCTTTATTAATGATATTCTTTATGGTTATGCCTGCCTTAATTGGAGGATTTGGTAATTTCCTTATGCCTTTAATGATAGGAGGTCCTGATATGGCATTCCCTAGATTAAATAATATTAGTTTCTGATTATTACCTCCTAGCTTAGTATTATTAATATTCTCTGCATGTATAGAAGGTGGTGCTGGTACAGGTTGAACTT**T**ATACCCTCCTTTATCAGGATTACAAAGTCACAGTGGACCTAGTGTAGACCTAGCTATATTCGCTTTACATCTTTCAGGGGTAAGTAGTTTATTAGGTGCAGTAAACTTTATAACTACAATAGCTAATATGAGAACACCAGGTATAAGATTACATAAATTAGCC**T**TATTCGGGTGAGCTGTAGTTATAACAGCTATCTTATTACTATTATCATTACCTGTGTTAGCCGGAGGTATTACAATGGTATTAACAGATAGAAATTTCAACACTTCATTCTTTGAAGTAGCTGGAGGTGGAGATCCAATATTATTCCAACATTTATTCTGATTCTTCGGTCATCCGGAAGTTTATATTTTAATTATTCCTGGTTTCGGTATAATTAGTACAACTATCTCAGCTAACTCAAGCAAACCTATATTCGGTTATATCGGTATGGTTTACGCTATGATGTCTATTGGAATCTTAGGATTTATCGTGTGATCACATCACATGTATACAGTAGGATTAGATGTGGATACAAGAGCTTACTTCACAGCTGCTACATTGATTATTGCTGTACCTACAGGAATTAAAATATTCTCATGATTAGCTACATGTTACGGAGGATCTATAAAAATGACACCATCAATGTTATTCTCATTAGGTTTCGTATTCATGTTTACAATTGGAGGATTAAGTGGTGTTGTATTAGCTAATGCATCACTTGATATCGCATTCCACGATACTTACTATGTTGTTGCTCACTTCCACTATGTTTTAAGTATGGGTGCAGTATTCGCAATGTTTGCTGGATGATATTTCTGAATACCTAAAATGTTAGGTTTAAATTACGACTTAACTTTAGCTAAAATACAATTCTGATTGTTATTCATAGGAGTTAATCTTACATTCTTCCCACAACACTTCTTAGGTTTACAAGGTATGCCTAGAAGAATAGGTGACTACCCTGATGCTTTTGCAGGATGAAACTTAATAAGTAGTGTTGGATCTATCGTAAGTGTAATAGCTGCTTGATTATTCTTATACATTGTTTACAAACAATTAGTAGAAGGTAAAGTAGCAAGTAGAAACCCTTGATTAACACCAGGATTCTATACTGACGTATTACAAGCTAACTTAAACAGATGTTACAGTAGTTTAGAATGAGGACTATCAAGCCCACCTAAACCTCACGCATTTGTAAGCTTACCTTTACAAAGTTAA

>COX2_NC_036382

ATGAAAAATTTATTAAACACATTTATATACTTAGACGCGCCTACTGCTTGAGGTATATACTTTCAAGATAGCGCTACTCCACAAATGGAAGGTTTAGTGGAATTACATGATAATATTATGTATTACTTAGTATTGATCTTATTTGCCGTAGGATGAATATTATTTTCTATAATAAAAAACTTTGCTGCAGCTAAAGCACCTATATCACACAAATACTTAAATCACGGAACTTTAATAGAATTAATATGAACTATAACACCAGCTCTTATATTAATACTTATAGCATTCCCATCATTTAAATTATTATATTTAATGGATGAAGTAAATGATCCTTCAATGTCAGTTCTTGCAGAAGGACATCAATGATATTGAAGTTACCAATATCCTGACTTTATAGATTCTAATGAAGAATTTATAGAGTTCGATTCATATATAGTACCAGAATCAGACTTAGAAGATGGTGGATTGAGAATGTTAGAAGTAGATAACAGAGTTATCGTTCCTGAATTAACACATGTTAGATTTGTTATAACATCTGGAGATGTTATACACTCTTTTGCTTGTCCTTCATTAGGTATAAAATGTGACGCATATCCTGGTAGATTAAATCAAGTATCAGTATTTATTAATAGAGAAGGAGTATTCTACGGACAATGTTCAGAAATATGTGGAATCTTACATAGTTCAATGCCTATAGTTATAGAATCTGTAAATATAGACAAATTTGTTCATTGATTATATAATGCTTAA

>COX3_NC_036382

ATGACTAATTTAGTAAGAAGTAATTTTCAAGATCATCCTTTCCATTTAGTGTCACCTTCTCCGTGACCACTATATACAAGTATATCTTTATTTTCTTTAACAGTAAATGCTGCATTATCTATGCATCTTTTCAATAACAGCTATATATTCTTCTATATGGCTTTAGTTACACTAGTAACATCTATGACTTTATGATTCAGAGATATAATCTCTGAAGGTACTTACTTAGGTAATCATACTTTAGCTGTACAAAAAGGACTAAATTTAGGTGTTATATTATTTATAGTATCTGAAGCTTGTTTTTTCGTAGCTATTTTCTGAGCATTCTTCCATAGCGCATTAACACCTACTGTTGAATTAGGTGCTCAATGACCTCCTATGGGTATAGATCCTGTTAATCCTTTCGAATTACCTTTATTAAACACAGTAATATTATTATCTAGTGGTGCAACAATTACTTATGCTCACCATTCTTTAATTAAAGGTGAAAGAAAAGGAGCTGTGTACGGTACACTTTTTACAGTATTATTAGCATTAATATTTACTGTATTTCAAGGAGTAGAATATAGCGTTTCTTCATTTACTATAAGTGACGGTGTGTTTGGTACATGTTTCTTCTTTGGAACAGGTTTCCATGGATTCCACGTTATTATTGGAACAATATTCTTAGCTGTAGGTTTATGAAGAATCTTAGCATATCACTTAACAGATCACCATCATCTTGGTTATGAAGGTGGAATATTATACTGACATTTTGTAGATGTTGTGTGATTATTCTTATATGTTTCAATGTACTATTGAGGATCTTAA

>NAD1_NC_036382

ATGAATTTACCTATAACTGTTATTTCAATTATTGAAAATCTATTATTAATGTTACCTGCATTATTAGTAGTAGCATACGTAACTGTGGCAGAAAGAAAAACTATGGCTAGTATGCAAAGAAGATTAGGTCCAAACGCTGTAGGTTATTACGGTTTATTACAAGCT**T**TTGCAGATGCTTTAAAATTAATTTTAAAAGAATATGTTGCACCTACACAAGCTAATTTAATACTATTTTTCTTAGGACCAATAGTAACATTAATATTTGCTTTATTAGGTTATGCTGTTATTCCTTACGGACCAGGGTTATCCTTAGGTGATATGGAATTAGGTATACTATTTATGTTAGCTGTATCATCTTTAGCTACTTATGGTATATTACTTGCAGGATGAAGTGCTAATAGTAAATATGCTTTCTTAGGATCTTTAAGAAGTACTGCTCAATTAATTAGTTATGAGTTAGTTTTAAGTTCAGTACTATTAATTATTATCATGATAACAAATAGTTTAAACTTAAATATAAATGTTCAATTTCAAAAGATAGTGTGATTAGCTTTACCTTTATTTTGTATATTAATAATATTCTTTATAGGTTCTGTAGCGGAAACTAATAGAGCTCCTTTTGATTTAGCTGAAGCCGAATCAGAATTAGTTAGTGGGTTTATGACAGAACACGCAGCTGTTATATTCGTTTTCTTCTTCTTAGCTGAATATGCAAGTATTGTTATAATGTGTATATTTATAAGTATCTTATTTTTAGGAGGTTATTTAGTGCAATTTGATTACATTTACGTATTTGATTCATTAAATTATATATATGCCTATTTATTTAATATAGAGTGAATTACATCTTCAGAATATTTTAAACTGAGAGGACTTTTAACTAGTTCTTCTATAGATGGACTATTATCTAGTTTAACTTTAGGTATTAAAAGTTCAATAATGGTATTTGTATTTATCTGAGTGAGAGCATCTTTCCCAAGAATTAGATTTGATCAATTAATGTCCTTCTGTTGAACTGTTTTATTACCAATACTATTTGCATTTATAGTATTAATCCCTTCATTATTATATATATTTGGAATATACTTTATAAATATAAGCTTATTTTAA

>NAD2_NC_036382

ATGATAATAATTTCAATTATAGCTCTTTTACTTTCTAATGCCGTTAATATAAGACGTGATATATCTATTCTATATAATAGAATAGCTATGCTTATTTTAATTTATTGTATCTTAAACGATTTATCCTCTTTAACTGTAGTAACCAAAGGTATAGGTCTACATGGAGGTTTATTGTTAGTTACAAATATTACACAAATATTTCATATATTCTTATTTATAGTAAGTATATTAATATTAACATTAACTAGTTTTTATCCTAGAAAAGTATGAGTATCTGAATACTCATCTATGAAAGATTTGTTATTATACAAATTTGTTTATTATAATACAAAAATAATAAATAAAATGGGAGAACATTTGAAAATAATAGAATATCCTTTAATATTATTATTTATAATAACAGGTGCAATATTCTTAATGTCAACTAATGATTTAGTTTCTATTTTCTTGGCAATAGAATTACAAAGTTATGGTTTATACATATTAAGTACTATATATAGAAATTCAGAATTATCTACTACAGGAGGTTTAATTTACTTCTTATTAGGTGGATTAAGTTCTTGTTTCATTTTATTAGGTACAGGTTTATTATATGCTAACTCAGGTAGTACTAGTTTAGATGGTTTATACATTATAACTAGCATAAGTGATATAAGCTCTACAGATTTATGATACAAACCTTACTATATTAATCTTTCATTAGTAATATTTACTATAGGATTCTTATTTAAGGTAAGTGCTGCACCCTTTCACTTCTGATCTCCTGATGTTTATGATGCTATACCTACTATAGTAACAGCATTTGTAGCTCTAATAGCTAAAATATCTATATTTATTTTATTATTACAATTAGTATATTATACTAATAATAGTTTCTCAGAAATGGGCTGAACATTTATATTATTAATGAGTTCTTTATTTTCATTAATAGTGGGAACAGTAGTGGGTTTAACTCAGTTTAGAATAAAAAGACTATTTGCTTATAGTACTATCTCTCATGTAGGGTTCTTATTATTAGCATTAGGTATATCTAGTATTGAATCTACTCAAGCATTTATTTTCTATTTAACACAATATACAATTAGTAATTTAAATGCATTTGTTATATTAATTGCTATAGGTTTCTCTTTATATTGCTATACAAGTGATAATAAAGAGCATGAAGAATTGGTGGATAAAACTAATTCTCCTATACAATTAGTAAGTCAATTAAAAGGTTATTTCTATATAAATCCTATATTAGCTTTAAGTTTTGCTATCACTATATTTTCATTTGTTGGTGTACCTCCTTTAGTAGGATTCTTTGGTAAACAGATGATATTAAGTGCAGCTTTAGATAAAGGTCTAGTATTTTTATCTTTAGTTGCTATATTAACTAGTGTTATAGGAGCAGTATACTATTTAAGTATAATAAAAGAAATGTTCTTCAGCAAACCTGATTATAAAGTTAATACTTTATTAGAAAATTTAGTATTAAAAGGTAATGTAATGGATAACAATAAAACAGTTATTAAAAATGTAAGTTTCAAATATAATAATATAGCTATATCAAGTCCAATATCTTTTGTTATATCTACTATAACATTAGTAATTTTATTATTTTTATTTATGAACAAAGAATGGCTAAGCATGGGTACCATATTGGTACAAATCTTATTTAATTATTAA

>NAD3_NC_036382

ATGAGTAGCGTAACTTTTCTTTTTATTCTTGTGTCTGTAATAACTATACTATTTTTAGCTCTTAATTTCATATTCGCACCTCATAACCCATATCAAGAAAAATATAGTATCTTTGAATGTGGTTTTCATAGTTTCTTAGGACAAAATAGAGCTCAATTTGGAGTAAAATTCTTTATTTTTGCTTTAGTGTATCTATTATTAGACTTAGAAATATTAGTAATATATCCTTTTGGTCTTAGTGGTTATGAAAACGGAGTATATGGTTTAATCATAGTACTTATATTCATAGGTATAATTACTGCCGGATTTGTATTTGAATTAGGTAAAAATGCATTAAAAATAGATAGTAGACAATCTTATAACTACTTTCATAAATCAAAAAGATTTATAAACACATTTATTGAAAATAAATAA

>NAD4_NC_036382

ATGTTATTATCCTCTTTATTAACTGTACCTGTGATAGGTACAATTATAGTATCTAGTATAGACTCATACAAAAAAGGTTCAGAAGTCTATACAAAAACAATCGCACTAGTAACAAGTGTAATAAACTTAATTATATCTTTAGTTATGTTTATATTATTTAATAATAGTACTAATCAATTTCAATATGTACAAGAACATTATAACGTACAATTATTTGATATTTATTTAGGTGTAGATGGTATATCTATATACTTTATATTATTAACTACAATAATAATGCCTATAGCATTGTTATCTAATTGAGATTCTATAAAAGAAAATGTAAAATCTTTCTTAATAATAATGTTATTATTAGAAACATTACTATTAGCTGTATTTATGGCATTAGACATAATGTTATTCTATGTTTTTTTCGAAAGTATATTACCTCCTTTATTTATATTAATAGGTATATTTGGATCGGATAACAAAGTAAGCGCTAGTTATTATCTATTCTTATATACATTATGAGGTTCATTATTCTTATTATTATCTATATTAAGTGTATCATCCATTATGGGTAGTACAGATTTTGATACTTTATTTAAATTAAACTTTGAATATAAAACACAAATATTCTTATTTATAGGAATATTTATAGCATTTGCAGTGAAAACACCAACAATATTCTTAAATAATTGACTATTAAAAGCTCACGTTGAATCTCCTTTAGGGGGAAGTATAGTTTTAGCTGCTATTGTATTAAAATTAAGCCTATATGGTATATTTAGATTAGTTTTACCTATATTACCCAAGGCATCTTTAGATTACACTTTTGTAATATATACTATAGCTGTAATTACTATAATATATGCTAGTTTCAGTACATTAAGAACAACTGACGTAAAAGAATTAATTGCTTATAGTTCTGTGTGTCACGCAGCTGTATATTTAATAGGAGTATTTAGTAATACAATACAAGGAGTAGAAGGAAGTATAGTCTTAGGATTAGCTCACGGATTTGTATCTAGCGGTTTATTTATATGTGCAGGTGGTATATTATACGATAGAACTGGTACTAGAAGTATATACTTCTATAGAGGTGCTACTCAACTAATGCCTATATTTGCTATATTATTCTTCATATTAGCTTTAGGTAATTGTGGTTCTCCTTTAACTTTAAATTTTGTAGGTGAATTTATGTCACTTTATGGAATAATTGAAAGATTACCGGTATTAGGAATATTTGCCTGTTCTTCTATAGTATTCTCTGCAGCTTATACTATTTATATGTTTAACAGAACAGCTTTCGGTGGTTCTTTCACTAGATTCTTGGAAGAAAGTGTATACGATGTTAACAAAAGAGAATTCACAATGTTATTTATATTAGTTGTATTTACTGTAGTATTCGGTATATACCCTTCATTAATATTAAACGTATTAGACTACTCTATGAATAGTTTAATATATAGTGTATAA

>NAD4L_NC_001715

ATGCTTTTAGAAATAATAACAGCTTATAAAATAGGAACAATCTTATTTTTAATTGGAATTTTAGGTTTCATTATCAATAGACAAAATATTCTTTTACTTATTATCTCTATTGAAATGACTTTATTAGCTATTAGTTTTATTATTATTTGTTCTGCTCTTTTCCTTGATGATTCTGCAGCAGCTTGTTTTTCACTTTATATTTTAGCTCTTGCTGGTTCAGAAGCTGCAATTGGTCTTTCACTTTTAGTTTTATTCCATAGATTTAGAGGATCAGTATTAATTTCAGCTTCTCGACAATAG

>NAD5_NC_036382

ATGTATTTAAGTATAATTATATTACCTTTATTAGGATCTATAGTATCCGGGTTTTTTGGTAGAAAAGTCGGTGTGACAGGAAGTCGTATATTAGGTTGTCTAAGTGTAATGACAACTACAATATTAGCTATTATTAGCTTCTTTGAAGTAGGATTTAATAATAATCCTATTTCTATTAATTTATTTAAATGATTAGATAGTGAATCATTTAATATAGCATGAAACTTTCAATTTGATAGTTTAACAGTATCAATGCTAATACCTGTATTAGTGATAAGTTCTTTAGTTCATTTATATTCTATAGGATATATGAGCCATGATCCACATAATCAAAGATTCTTTAGCTATTTAAGCTTGTTTACTTTTATGATGATTATATTAGTAACAGGTAATAATTATCTATTAATGTTTGTAGGATGAGAAGGTGTTGGAGTTTGTTCATATCTTTTAGTTAGTTTCTGATTCACTAGAATTGCAGCTAACCAAAGTTCTTTATCTGCATTTTTAACTAATAGAGTAGGGGATTGTTTCTTAACAATGGGTATGTTTGTAGTATTATGATCTTTAGGTAATTTAGATTATAGTATAGTATTCTCAGTAGCCCCCTATATTAACGAAAATATTATAACAATTATAGGTATTTGTTTATTAATAGGGGCTATGGCTAAAAGTTCTCAAGTAGGTCTTCACATATGATTACCTATGGCCATGGAAGGTCCTACACCTGTATCTGCGTTAATACACGCGGCTACAATGGTTACAGCAGGAGTGTACCTATTAATACGTTCATCTCCTTTAATAGAATATAGTTCAACTGTTTTATTAATATGTCTTTGATTAGGTGCTATCACAACAGTATTTAGTTCTCTTATAGGTTTATTCCAACAAGATATTAAAAAAATTATTGCTTATTCTACTATGAGTCAATTAGGTATGATGGTTATAGCTATAGGTTTATCTTCTTATAATATTGCCATATTCCACTTAATAAATCATGCTTTCTATAAAGGATTATTATTCTTAGGGGCAGGTGCTGTAATACACGCTGTGGCAGACAA**T**CAAGATTTAAGAAGATATGGAGGATTAGTTTCATTCTTACCTTTAACTTATACAGTGATATTAATAGCTAGTCTTAGTTTAGTTGCTTTCCCTTTTATGACAGGGTTCTATAGTAAAGATTTTATATTAGAATCTGCTTATGGTCAGTATTGCTTTAGTAGTATAAACGTTTACTTTATAGCAGTAATAGGTGCAATATTCACTACATTATACTCAGTGAAAGTTATATACTTAACATTCTTAGCTAATCCTAATGGACCTGTAAATTATTATAAAAATGCTCATGAAGGTGATATCTTCTTAAGCTTACCTTTAGTTATATTAGCCATATTTTCTATATATTTCGGATTTTTAACTAAAGATATATTTATCGGTTTAGGTTCAGGATTCTTTACAGATAATAGTATATTTATTCATCCTATGCATGAAATATTAATAGATACAGAATTTGCTGTACCAACATTGTTTAAATTACTTCCTTTAGTATTCACAGTATCATTTTCGGCTTTAGCTATTATATACCCAGAATTTATGCCTAGTGCCATAACTAATTTCAAACTATCTAGCTTAGGATACTACGTATTTGGTTTCTTTAATCAACGTTTCTTAGTAGAATTCTTCTATAATAAATTTATAGTTAATACAGTTCTAGATATAGGAGGTCAAACAACTAAAGTGTTAGACAAAGGTAGTATAGAATGAGTTGGTCCTTATGGTATGGGAGTAATGTTAACTAAAACAAGTAAAACAATATCTGGTTTAAGCAAGGGAGTTGTTACTGATTACGCTCTTTATATATTAATAGGAGTTTGTTTCTATTTATCAATATTTACTTTTGTTTCAATATTCTTTGACTTAGTTAATTCTATTACAGTATCTTGTATAATAGTTTTAATAGGTATTAGTAACTATATTATATCAAGTAAAGACAATGAGAACAAAGTCATCTAA

>NAD6_NC_036382

ATGAATCAATTATTGGCCATTTATGATATTTTATCAAACGGGTATACAGTAGAATATTTAGATGTTTTAAGTGTAATAGCATTATTATTTGGGATATCGGTTATAATCAACAAAAATCCTATAGCGTCTCTTTTATCTTTAATAGGATTATTTGCATCTATTTCTGTTTATTTAATATTATCAGGATTAACTTTTATAGGTTTCTCTTATTTAATTGTATATATAGGGGCAGTCTCTATATTATTCTTATTTATTCTGATGTTAATTAATATAAGAACAAGCGAGTTACAAAGTAACAACTGAAATAGTATTCCTTTAGCACTATTTGTAACAATATTATTGAATTATGCATTATTTCAATTATTACCTTATTATATAGCTATAATAAATAATTATAATAGTAAAATAAGTAATTTAATATATTACTTACCTACAAGTAAATACAATGATATTATAACTCATATAAATAAAACTGTAGATGTAAACACATCTAATGTTATGTTTGTAACAAGTAATAGTTGAGATGGAAATATGACAGAAACAAGCCATATTTCTACAATAGGAAATATATTATATACTTCTTACAATATGTGATTATTTATTGCTAGTATGATATTATTATTAGCAATGGTAGGAGCTATTATCATAACTATAAAACAAGAACGTAATAGGGAAATTAGTTCAATTGGCGGAACGTTTGTTTTACACACAAAATGTTAA

>rps3_NC_036382

ATGAAAGTATTTAATAATAACTTAAAAAATATACCTAAAACTATTGCTTTAAGAAATAAAGTAGGAGATATAGGGAAAACAAAATATTTACCCTCTTTCTCTAAAGAATGAAAAAATATTGTTTATTCTTATAATAAAAATAATTTAAAGAATATACCCGTTAATGATGTAAATATTAATAAAATAATACAAAGTTATTTTAATTTATATTTCAAAGATCATAAATATGTAGGTTCTAAGAAATTTATATTACTTAGAAGAAGACGTAATTTTTTAAGAAGAATATATGTAAGTAATGCGGAAATTAAACATACGAATAATAAAGCGATAATTACTTTATTCACTGTAAATAGAGAAAAGAAAATATTAAAAAAGAAATATTTAAAAATAAATAAAAAAGTAAGTAAAAATTTAATAAAACGTTATTTCTTATTATATAAAAATAATATAACTAGAATATACGAAATATTAAATAAACAGAAAAATGAATATGCTTTTATATCAGATAAAATTTCAAAAAGAAAATTTTTAACTTATAAATTAGAATATTTAAACAAATTTATAGAATTAAAGAATCTTTACTTAAAGAAGGTATGAAGTATTATTATTAGCAAATATTGAAAAACATATTTGAGATTATTAAGAAAATATGATTTAATGTATTCTCTTAATCAATATAAATTTAATAAACAAATGCTTTTACCTATTTTAAGTAATATATTAAACAAAATAATAGGAAAGAAAGTAGAATATAATATAATAAATCTAAAATCTATTGCATACAACACAGATCTTTTTACTAATGCTTTATCTTTAAAATTAAAAAAAAAGAGAATGAATTATATAAAAAGTATGTTTAGTATATTAAATAGAGCTTATTTACCTAAAATTAATACAATAAAAGAAAGAACTTTAGCTAAAAATGTTGATTTATTTTTAGATAAATATAAAGATTTGAAAATAATTTCTAATCTGGGCGCAAGCTCTAATAATAATTTAGATAAATTATTAGGTGATTCTTCTAATACTAAAGAAGTTCATAACACAATATACAACTCTATAGGTTATAAAAACATGGGAGGTATAAGATTAGAAGTTAAAGGTAGATTAACTAAACGTTATAGAGCAGATAGATCTATATATTCACTAAAATGAAAAGGTGGATTAAAAAATGTAGATTCATCATTCAAACGTTTAAGTTCAGTCTTATTTAGAGGAAACTCTAAATCAAATGTATCTTATTCATTATCTAACTCAAAGCGTCGTATTGGGGCTTTTGCAGTAAAAGGTTGAATAAGTGGTAAATAA
