# Supplementary material for: Proposal of a new nomenclature for introns in protein-coding genes in fungal mitogenomes
Source: IMA Fungus. 2019 Oct 10;10:15. doi: 10.1186/s43008-019-0015-5 (PMC7325650; doi:10.1186/s43008-019-0015-5)
Supplement: Supplementary file 2 — Intron insertion sites for 22 common introns. Exon sequences of cob, cox1, cox2, nad1, and nad5 of different fungal taxa plus few non-fungal taxa were aligned by MAFFT, and visualization of the aligned sequences was performed using ESPript 3.0 (Robert and Gouet 2014) under default settings. Refer to Tables 1 and 2 for organisms represented by accession numbers, and the accession numbers of non-fungal taxa are marked in red boxes. Insertion sites of introns are shown using upward arrows. For phase 0 introns, conserved amino acids before and after insertion sites are listed. The amino acid glycine (G) is frequently seen before insertion sites of phase 0 introns. For phase 1 or 2 introns, conserved amino acids at insertion sites are given, and corresponding triplet codons are marked by a horizontal line. (PPTX 2235 kb) [file 43008_2019_15_MOESM2_ESM.pptx]

## Slide 1
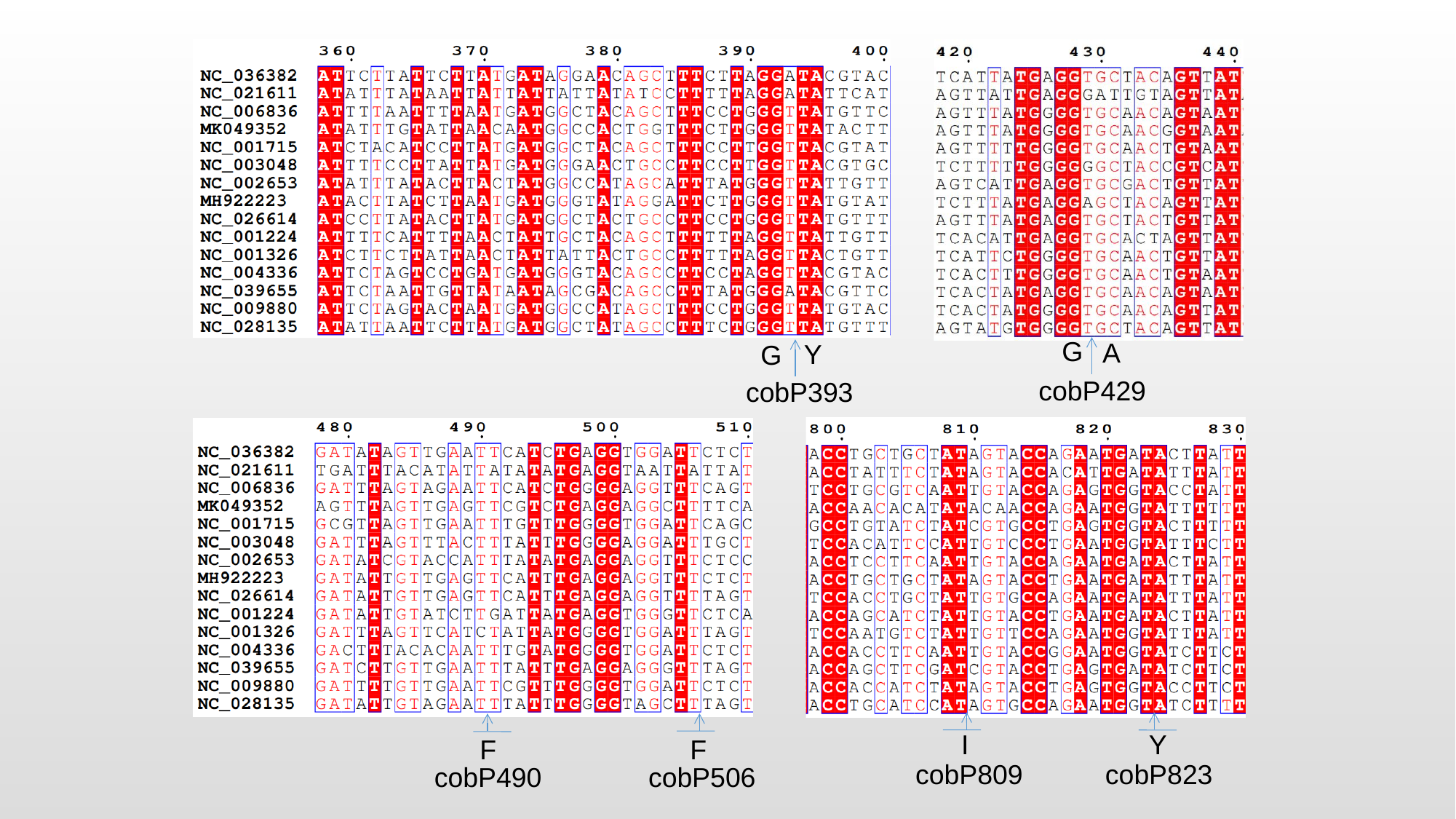

G
A
Y
G
cobP429
cobP393
I
Y
F
F
cobP809
cobP823
cobP490
cobP506

## Slide 2
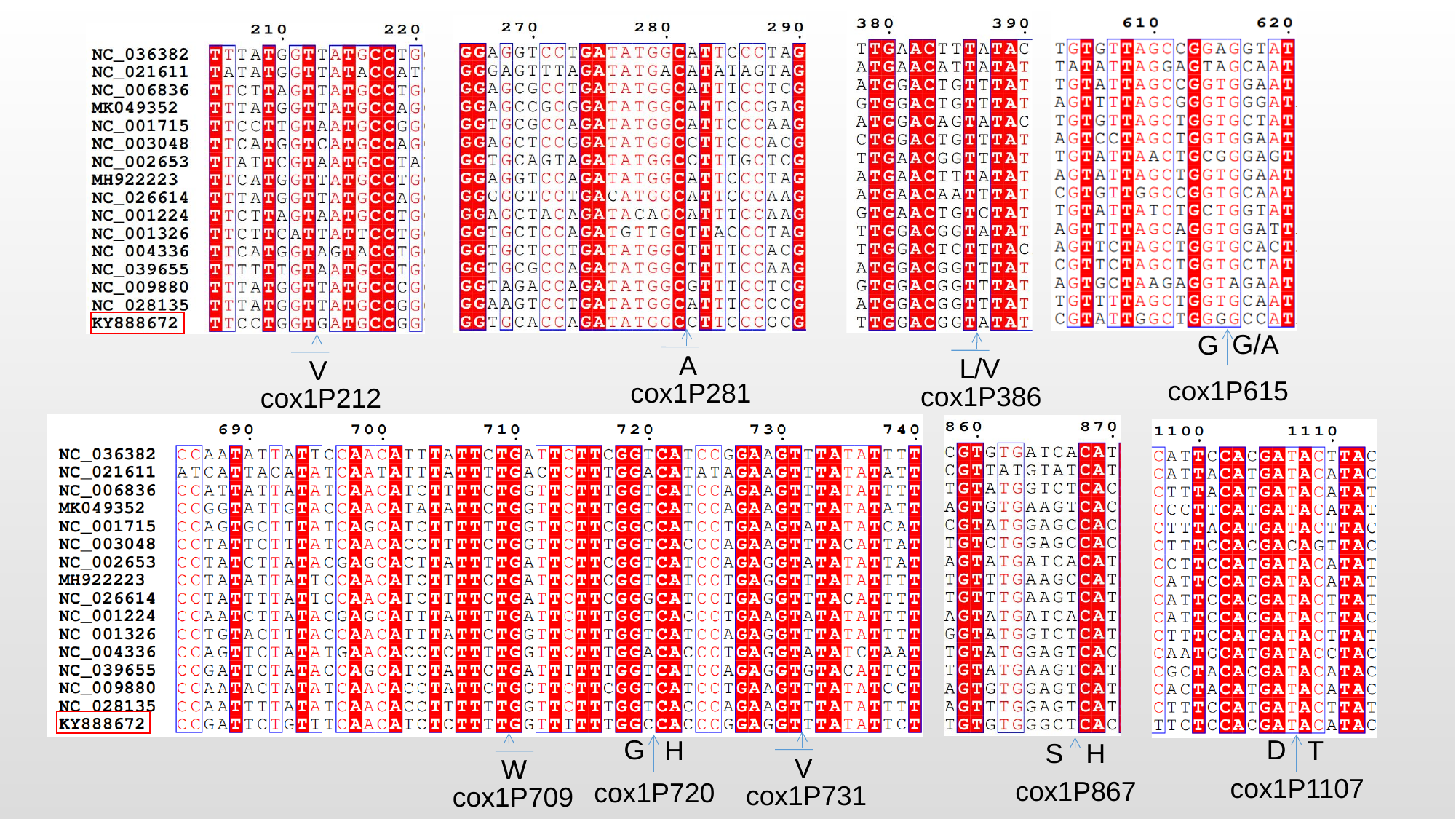

G/A
G
A
L/V
V
cox1P615
cox1P281
cox1P386
cox1P212
G
D
H
T
S
H
V
W
cox1P1107
cox1P867
cox1P720
cox1P731
cox1P709

## Slide 3
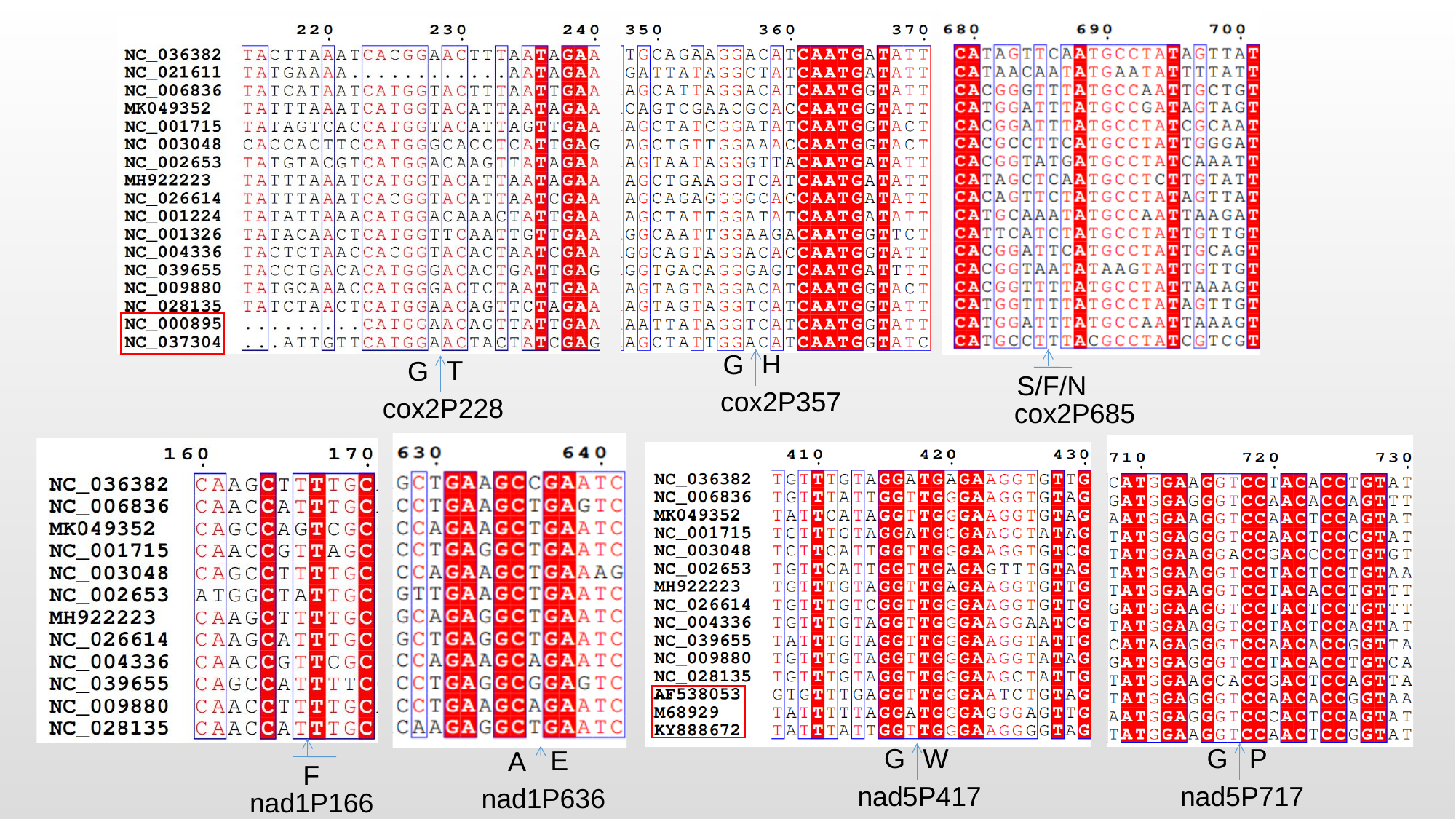

H
G
T
G
S/F/N
cox2P357
cox2P228
cox2P685
W
P
G
G
E
A
F
nad5P417
nad5P717
nad1P636
nad1P166
